# Low Socioeconomic Status Leading to Unsafe Abortion-related Complications: A Third-world Country Dilemma

**DOI:** 10.7759/cureus.3458

**Published:** 2018-10-16

**Authors:** Hania Zafar, Hina Ameer, Rahma Fiaz, Saad Aleem, Shazia Abid

**Affiliations:** 1 Obstetrics and Gynecology, The Indus Hospital, Lahore, PAK; 2 Obstetrics and Gynecology, Air Force Hospital, Shorkot, PAK; 3 Family Medicine, District Headquarter Hospital, Sahiwal, PAK; 4 Orthopedics, Nishtar Medical College and Hospital, Multan, PAK

**Keywords:** unsafe abortion, socioeconomic status, third world country

## Abstract

Introduction

An unsafe abortion is a persistent, preventable dilemma. It is a procedure where an unintended pregnancy is terminated either by untrained individuals, or in an environment not meeting medical standards, or both, as defined by the World Health Organization (WHO). It endangers women in developing countries, where abortion is restricted either by law and culture or legally permitted but not easily accessible. Induced abortions are usually performed by unqualified and untrained individuals or are self-induced. Such incidents take place in unhygienic conditions and involve inappropriate methods or administration of medications. Even if carried out by medical experts, a clandestine abortion carries an additional risk, medical coverage is not immediately available in an emergency and the woman may not receive appropriate post-abortion attention. Induced abortion-related complications happen and the woman may hesitate to seek medical care. Unsafe abortion-induced complications contribute a major burden, such as increased hospital stay, drug costs, and an unusual delay of other operations on gynecological services in developing countries. The purpose of this study was to seek an association between low socioeconomic status and complications related to unsafe abortion.

Materials and methods

A total of 296 female patients of child-bearing age presented between 2012 and 2015 in the emergency department (ED), Nishtar Hospital, Multan, after an unsafe abortion, were included. Spontaneous miscarriages and abortions cases carried out on legal or medical grounds were excluded. Patient or their attendants (who usually present the real picture of incidents leading towards unsafe abortion) were interviewed for determinants leading to unsafe abortion. A detailed clinical assessment of the patient was done and complications like hemorrhage, uterine perforation, and bowel perforation were recorded along with basic demographic information such as age, gestational age, parity, and weight.

Results

There were 296 female patients in the study with a mean age 28.391 ± 4.57 (Range: 13-40 years). In a majority of patients, gravida and parity were 5-6. The mean weight was 60.283 ± 9.31 kilograms and the mean gestational age was 7.733 ± 2.45 weeks. The determinant in the shape of poor economic status was 71.6%. Hemorrhage was seen in 30.1% of the patients followed by uterine perforation (49.3%) and bowel perforation (45.6%).

Conclusion

Our results indicate that unsafe abortion is a major cause of maternal morbidity, mostly because the service is being sought from untrained healthcare providers in unhygienic conditions secondary to poor socioeconomic status. Since maternal morbidity due to unsafe abortion is a violation of a woman's basic human right: the right to life, there is a dire need to prevent these unwanted complications by improving the quality of the family planning program and providing safe abortion services.

## Introduction

Every year, an estimated 210 million women throughout the world become pregnant and about one in five of them resort to abortion. Out of 46 million abortions performed annually, 19 million are estimated to be unsafe [1]. The World Health Organization (WHO) defines unsafe abortion as a method of terminating an unwanted pregnancy either by untrained individuals, or in an environment with compromised medical standards, or both [2]. The incidence of unsafe abortions remains high in the developing world; the highest rates are observed in Africa, Latin America, and the Caribbean, followed by South and South-East Asia. It has been estimated that approximately 68,000 women lose their lives every year as a consequence of unsafe abortions and 5.3 million suffer a temporary or permanent disability [3]. The public health burden is greatest in the developing world. Saleem and Fikree found that women in low-socioeconomic settlements in Karachi opt for abortions rather than using modern contraception to attain their goal of small family size [4]. The majority of couples who seek an abortion had four or more children. In the developing world, including Pakistan, the morbidity and mortality associated with unsafe abortions put a significant impact on the health, financial, and social aspects of the healthcare system [5]. The objective of this study was to highlight an association between poor socioeconomic status and complications related to unsafe abortion.

## Materials and methods

A total of 296 females reported between 2012 and 2015 at Gynecology Unit 1, Nishtar Hospital, Multan, Pakistan, after an induced abortion from outside with a mean age and standard deviation of 28.39 ± 4.60 years were included in the study. Spontaneous miscarriages and abortions carried out on legal and medical grounds were excluded. Patients or their attendants were interviewed for determinants leading towards unsafe abortion. A detailed clinical assessment was performed and complications like hemorrhage, uterine perforation, and bowel injury were recorded along with basic demographic information, such as age, gestational age, parity, and weight. Low socioeconomic status was assessed on the basis of education, employment, and monthly income (Illiterate, unemployed, and monthly income less than 100 USD per month were categorized as low socioeconomic status). Informed consent was obtained from the patients and permission for the study has been granted by the ethical review board (IRB) of Nishtar Medical College and Hospital, Multan.

A statistical analysis was carried out using the SPSS software (SPSS version 20.0; SPSS Inc., Chicago, IL, USA). Continuous variables were stated as mean ± standard deviation (SD) and categorical variables were computed as frequencies and percentages. Categorical variables were compared using the chi-square test or Fisher's exact test (when necessary). The continuous variables were compared using the independent t-test. Statistical significance was defined as a two-tailed p-value of 0.05.

## Results

The majority of the participants had a poor socioeconomic status (71.6%). The average frequency for gravida was 4.90±1.80 with gravida 5 and 6 comprising more than 50% of the women seeking unsafe abortions. Furthermore, uterine perforation was observed to be the most common complication (49.3%) followed by bowel injury (45.6%) and hemorrhage (30.1%), as shown in Table 1. The age range in this study was from 13 to 40 years with a mean age of 28.391 ± 4.57 years. The mean weight was 60.283 ± 9.31 kilograms and the mean gestational age was 7.733 ± 2.45 weeks. Additionally, uterine perforation and bowel injury showed a statistically significant association with poor socioeconomic status (p-value 0.001), as shown in Table 2. A comparison of hemorrhage, uterine perforation, and bowel injury with poor socioeconomic status is reported in Tables 1-2.

**Table 1:** Descriptive statistics *standard deviation; **kilogram

Variables	Statistics
Age (mean ± SD*)	28.39 ± 4.60
Weight (kg**) (mean ± SD*)	60.28 ± 9.32
Gravida (mean ± SD*)	4.90 ± 1.80
Parity (mean ± SD*)	4.80 ± 1.70
Gestational age (mean ± SD*)	7.73 ± 2.45
Poor socioeconomic status (N (%) )	212 (71.6%)
Hemorrhage (N (%))	89 (30.1%)
Uterine perforation (N (%))	146 (49.3%)
Bowel injury (N (%)) ​​​​​​​	135 (45.6%)

**Table 2:** Linking of association between poor socioeconomic status with explanatory variables *standard deviation; **kilogram; ***Pearson chi-square (p-value)

Variables		Socioeconomic status		p-value
		No (84 (28.4%))	Yes (212 (71.6%))	
Age (mean ± SD*)		28.70 ± 4.44	28.27 ± 4.64	0.46
Weight (kg**) (mean ± SD*)		59.05 ± 8.50	60.77 ± 9.60	0.15
Gravida (mean ± SD*)		4.75 ± 1.58	4.96 ± 1.87	0.36
Parity (mean ± SD*)		4.68 ± 1.51	4.79 ± 1.73	0.60
Gestational age (mean ± SD*)		7.58 ± 2.15	7.79 ± 2.56	0.51
Hemorrhage				0.002***
	Present	14 (16.7%)	75 (35.4%)	
	Absent	70 (83.3%)	137 (64.6%)	
Uterine perforation				0.001***
	Present	21 (25.0%)	125 (59.0%)	
	Absent	63 (75.0%)	87 (41.0%)	
Bowel injury				0.001***
	Present	14 (16.7%)	121 (57.1%)	
	Absent	70 (83.3%)	91 (42.9%)	

## Discussion

Lack of training, unfamiliarity with treatment modalities, expired drugs, broken equipment, sporadic electricity and water, and transportation challenges all threaten the health of women grappling with unsafe abortions [6]. Perhaps the greatest danger of all is indifference—or overt disdain.

 In Pakistan, the law concerning abortion is restrictive, where induced abortion can only be allowed in order to save the lives of women or to provide the necessary treatment early in pregnancy. Since a ma­jority of unsafe abortions are conducted without disclosure, it is, therefore, difficult to determine the exact numbers of unsafe abortions. Information regarding abortion is derived from studies on patients admitted to hospitals due to complications of abortion [7].

The majority of women with an unwanted pregnancy usually contact close friends or go to traditional healers. They may otherwise find and take the non-recommended abortifacients, such as castor oil or some forms of herbs. This may either prove to be successful or result in disastrous situations such as hemorrhage, shock, infections, sepsis, perforations, organ failure, chronic pelvic inflammatory disease, infertility, or even death. The unethical and unlawful practice by healthcare providers or traditional untrained and unskilled healers, supplemented by poor hygiene standards and lack of expertise, have caused major complications.

The international statistics on unsafe abortion have remained static between 1995 and 2003 (15/1000). The overall abortion rate has gone down but the proportion of unsafe abortion has increased. A majority (95%) of the abortions in Africa and Latin America and a significant proportion (60%) in Asia (excluding the Far East) are performed in sub-optimal conditions [8]. The total first abortion rate in Australia is 29% while in Nigeria, 18-25/1000 of known pregnancies undergo abortion [9]. Termination of pregnancy, either legal or illegal, is associated with complications. The mean age of our patients was 28.3 years, matching a study carried out in Pakistan and India [10-11]. Most of the sufferers were older women, in contrast to western studies, where the majority are teenage girls [12-13].

The commonest complication encountered was hemorrhage in 30.1% cases. This is also common in legal abortions where the incidence is 1/1000 [14]. The second-most-common complication was bowel perforation (45.6%) in our series, matching the 42.8% reported in a study carried out in the west of Pretoria [15].

The most drastic of the complications is uterine perforation (49.3%), with or without intestinal involvement, similar to the trend in the literature [15-17].

Most of the abortions occur in the first trimester, during which the uterus is placed in the pelvis in proximity to the sigmoid colon and rectum, making them more susceptible to injury [18]. With the increasing size of the uterus, the small bowel becomes more prone to injury during unsafe abortion [19].

The most important reason for women to seek induced abortions seemed to be the fact that obstetricians and gynecologists usually refuse to perform terminations either due to the social stigma associated with abortion or having inadequate knowledge about the abortion law.

Before 1997, abortion was only allowed to save the life of the woman, but in 1997, the law was amended to allow abortion in early pregnancy not only to save the life of the woman but also to provide necessary treatment [20]. Moreover, doctors are known to refuse termination on the basis of their own personal beliefs (either ethically or religiously driven). It has been suggested that doctors who feel an ambivalence about providing abortion services undergo a values clarification and attitude transformation exercise; hence, they should be able to separate their personal beliefs from their professional responsibilities of saving women's lives [21]. According to the Federation of International of Gynecologists and Obstetricians (FIGO) Resolution 2006, doctors who refuse termination on the basis of conscientious objection are still under ethical obligation to refer women to safe services and provide the service themselves in case of an emergency [22].

In 2012, almost 6.2 million women have been treated for post-abortion complications in both the public and private sectors. This rate is quite high when compared with other neighboring third-world countries [23]. The poor state of public health facilities in Pakistan, apart from the rapidly increasing population, has exerted pressure on state institutions. This has allowed the private sector to step in and provide health care to the majority of the population. In our experience, most of these mismanaged cases were treated by unskilled individuals, such as midwives who are not even licensed to handle such cases. The low socioeconomic status of people has urged them to seek cheap medical services, which has contributed to the increased frequency of complications related to abortion. The workload on the public sector and the cost-related issues of good quality in the private sector have also encouraged people to look for alternate options. Even if treated by professionals, one cannot be sure about the standard of facilities that the private sector offers, particularly in rural areas.

There should be a nationwide campaign from the policy makers and service providers to make people aware of the use of contraceptive pills. Standards of post-abortion care are seriously lacking in the country as per WHO standards. The mushrooming growth of these sub-standard private rural centers providing cheap facilities to terminate unintended pregnancies has resulted in a significant rise in the number of complications such as hemorrhage, bowel injury, and uterine perforation. It has a significant impact on the burden of patients that the public sector receives from across the country. There is a dire need to raise awareness and facilitate women to seek unintended abortion-related care in the right hands, keeping in view the low socioeconomic status of most of the population.

## Conclusions

Our results indicate that low socioeconomic status is associated with unsafe abortion-related complications and maternal morbidity, mostly because the service is being provided by untrained healthcare providers under unhygienic conditions in sub-standard private setups. Since maternal morbidity secondary to unsafe abortion is a violation of the women's basic human right - the right to life - there is an urgent need to prevent these unnecessary complications by improving the quality of the family planning program and providing safe abortion services irrespective of the socioeconomic status of the population.
